# Twist in the Bowel: A Multimodality Radiological Imaging Spectrum

**DOI:** 10.34172/mejdd.2024.381

**Published:** 2024-04-30

**Authors:** Siddhi Chawla, Lalendra Upreti, Thaihamdao Halflongbar

**Affiliations:** ^1^Department of Radiology, Sardar Patel Medical College Bikaner, Rajasthan, India; ^2^Department of Radiology, Rajiv Gandhi Super-Speciality Hospital, Tahirpur, Delhi, India; ^3^Department of Radiology, Maulana Azad Medical College, Delhi, India

**Keywords:** Volvulus, Abdominal emergency, Computed tomography, Abdominal radiology

## Abstract

Volvulus affecting the gastrointestinal (GI) tract is one of the common causes of recurrent pain in the abdomen, and often, patients present with non-specific abdominal pain associated with nausea and/or vomiting. A high degree of suspicion is required at the clinician’s end to suspect this diagnosis, which is usually confirmed by imaging using radiographs, fluoroscopic evaluation, and computed tomography. Familiarity of the clinician and radiologist with the imaging appearances of these emergent conditions on various imaging modalities is quintessential to avoiding life-threatening complications like bowel ischemia or perforation, which are associated with delayed or missed diagnosis. Our article describes the clinical features and classical imaging of the various types of volvulus affecting different bowel segments in the entire GI tract.

## Introduction

 Volvulus involving the gastrointestinal (GI) tract is a common cause of acute abdominal pain.^1^ It can involve any site along the entire GI tract i.e stomach, small bowel, small intestine, cecum, transverse colon and sigmoid colon. Sigmoid volvulus is the most common of these bowel segments and accounts for 60%-75% of cases of intestinal volvulus.^1^ Patients commonly present with non-specific complaints, including variable degrees of acute onset pain associated with bloating, nausea, and/or vomiting. It is rarely diagnosed just on the basis of clinical symptoms, and hence, radiologists play a vital role in not only diagnosing but also in evaluating the probable etiology leading to volvulus. They also prognosticate the patient based on the imaging findings. For diagnostic evaluation, plain radiography, fluoroscopy, and computed tomography (CT) are commonly used.

 Volvulus occurs when a loop of the bowel twists around itself with the mesentery that supports it causing obstruction of the bowel at the point of twisting.^2^ If the patient presents late, symptoms like fever, constipation and bloody stools may occur. This occurs because the mesentery becomes extremely tightly twisted such that its blood supply is cut off, resulting in an ischemic bowel.^2^ At this stage, there can be a paradoxical relief from pain, as due to bowel ischemia, there is damage to the nerve endings supplying the ischemic bowel, which relieves the pain, but the patient’s blood counts continue to increase as infarcted acts as a breeding ground for infection.

 Although there is a difference in age at which a particular volvulus occurs (Table 1),in adults, the sigmoid colon is the most affected, followed by the cecum, while in children, the small intestine is more commonly involved.^3^ There are various risk factors that are common at all ages, including intestinal malrotation, Hirschsprung disease, and abdominal adhesions (Ladd’s bands or postsurgical). In children, most cases of volvulus are primary, i.e., due to congenital causes like abnormally long or short mesentery of affected organs, ladds band, or malrotation.^4^ High fiber diet, postsurgical adhesions and chronic constipation are identified as risk factors only in adults.^5^

**Table 1:** Age-wise predominant incidence of different types of volvulus

**Children**	**Adults**
Mesenteroaxial gastric volvulus Midgut volvulus	Organoaxial gastric volvulus Cecal volvulus Sigmoid volulus

## Gastric Volvulus

**Figure 1:**
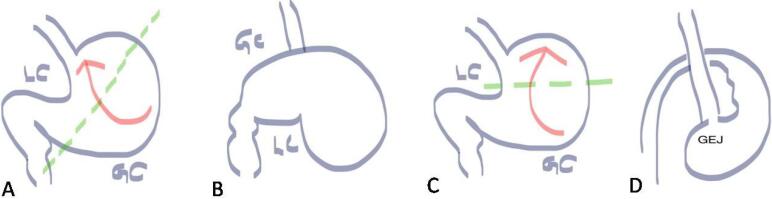
Diagrammatic representation of the organoaxial (images A, B) and mesenteroaxial (images C, D) types of gastric volvulus. The dotted green line in images A and C demonstrates the axis of rotation. The red arrows demonstrate the point of rotation of the bowel. GC: Greater curvature; LC: Lesser curvature; GEJ: Gastroesophageal junction

 The stomach is not a common site for volvulus as the twist of the stomach on its mesentery should be at least 180° to cause bowel obstruction. The clinical triad for identifying gastric volvulus is known as the Borchardt triad. It consists of sudden epigastric pain, intractable retching, and inability to pass a nasogastric tube into the stomach.^6^ Gastric volvulus is divided into two main subtypes: organoaxial (Figure 1A, B) and mesenteroaxial (Figure 1C, D). Organoaxial volvulus is more common than mesenteroaxial volvulus and accounts for 2/3^rd^ of the cases of gastric volvulus. The major differences between the two are highlighted in Table 2.

**Table 2:** Differences between organoaxial and mesenteroaxial types of gastric volvulus^6,7^

	**Organoaxial**	**Mesenteroaxial**
Age	Adult	Children
Etiology	Post-trauma, associated with paraesophageal hernia (adults)/ Bochdalek hernia (children)	Less commonly associated with diaphragm defects
Axis of rotation	Along the long axis of the stomach	Along the short axis
Antrum	Rotates anterosuperiorly	Above gastroesophageal junction
Fundus	Rotates posteroinferiorly	Same place
Severity	complete ( > 180º) present with obstruction or ischemia*	usually incomplete, < 180
Treatment	Surgical- if symptomatic	Surgical – if symptomatic

* < 180 degrees called organoaxial position. These patients are usually asymptomatic.

**Figure 2:**
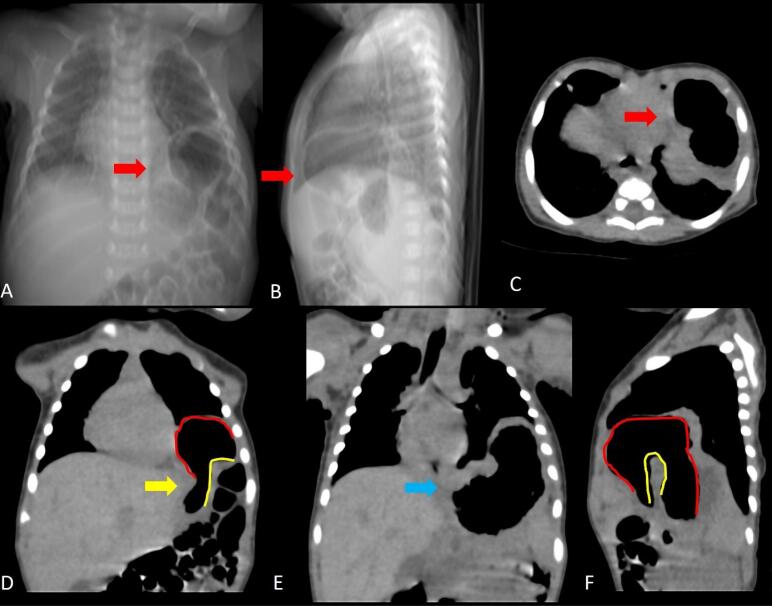
Organoaxial type of gastric volvulus (child): A 12-day-old baby presented with regurgitation of food for 2 days. Chest radiographs anteroposterior (A) and lateral (B) show a defect in the anterior left hemi-diaphragm herniation of the stomach ( red arrows in A and B). A non-contrast CT was done for the patient to look for the size of the defect. Axial, coronal and sagittal sections showed herniation of the stomach (red arrow in C) with reversal of greater and lesser curvature (red and yellow arrow respectively in D and F). The anterior section shows a pyloric end (D), and the posterior section shows the gastroesophageal junction at a normal location in an infra-diaphragmatic location. The final diagnosis intraoperatively was diaphragmatic hernia with stomach as content and organoaxial type gastric volvulus

**Figure 3:**
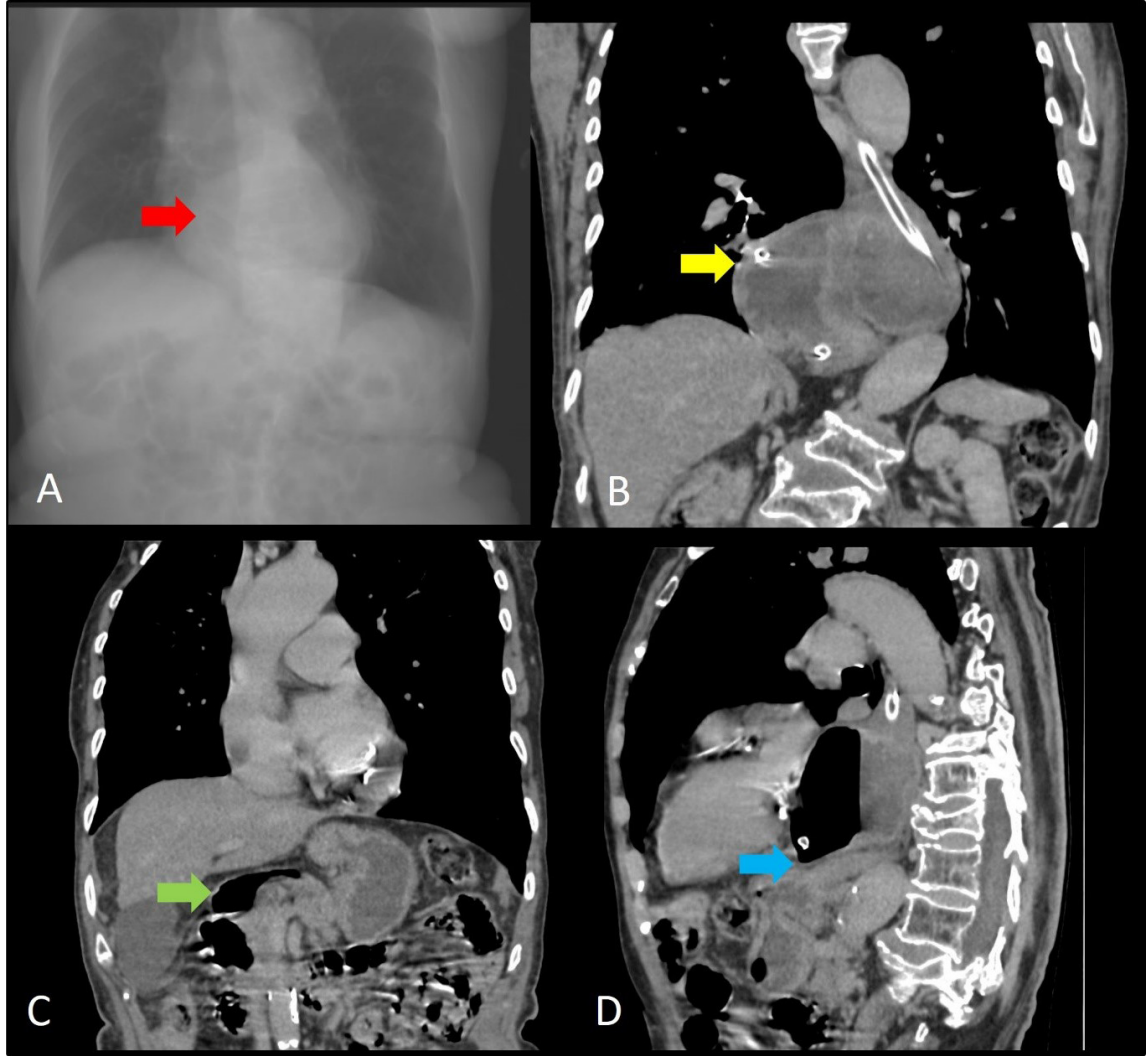
Organoaxial type gastric volvulus (adult): An 85-year-old male patient presented with acute abdominal pain with retching and vomiting. Chest radiograph shows a lucent radiopacity overlying the heart shadow (red arrow in A) with non-visualization of stomach bubble in the left subdiaphragmatic location. Coronal and sagittal sections of CT show a sliding hiatus hernia of the stomach with Ryle’s tube coiled within the herniated part of the stomach with reversal of greater and lesser curvatures (yellow arrow in B). Pylorus is seen in the abdomen (green arrow in C). Diaphragmatic defect at hiatus is seen by the blue arrow in D. Final diagnosis intraoperatively was sliding hiatus hernia with organoaxial gastric volvulus in herniated part

**Figure 4:**
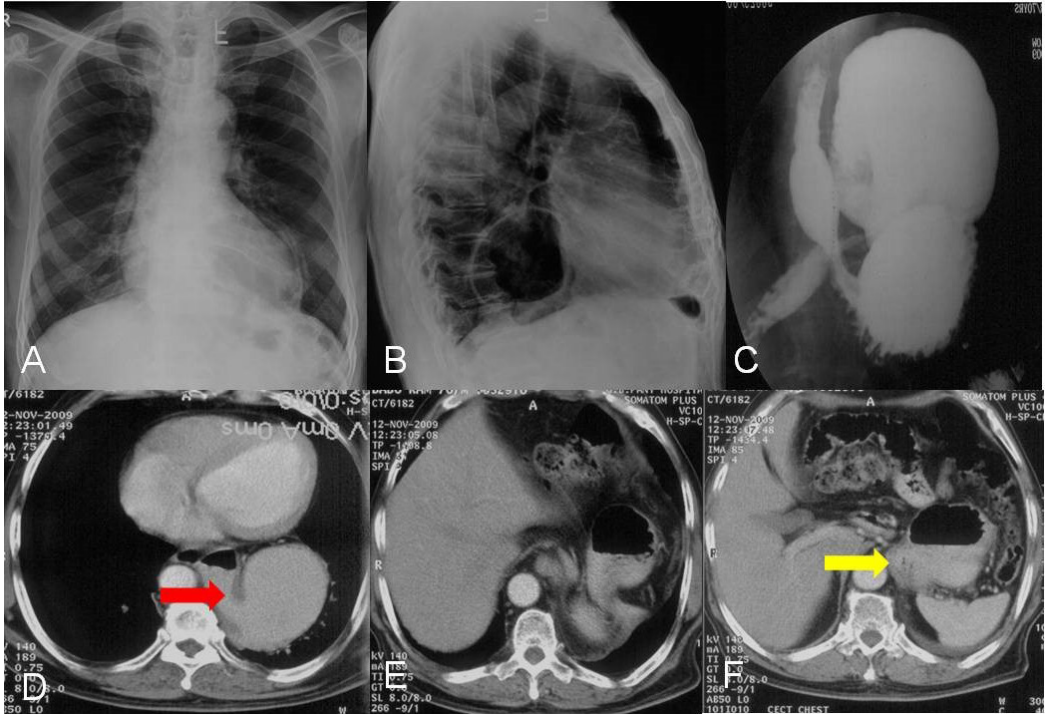
Mesenteroaxial type of gastric volvulus: A 73-year-old patient presented with constipation, abdominal pain with distension, and non-passage of flatus/stools. The frontal and lateral radiograph of the chest shows a well-defined air-filled lesion in the middle mediastinum. Spot barium meal image shows the antrum of the stomach herniating above the level of the diaphragm with the fundus at its normal position. Contrast is seen to pass distally. Axial contrast-enhanced tomograms confirm the findings of the barium study. The pylorus (red arrow in image D) is seen above the level of the diaphragm and gastroesophageal junction (yellow arrow in image F) is seen at its normal location

 Plain radiographic findings in gastric volvulus may include herniation of a large portion of the stomach above the diaphragm with air-fluid levels (Figure 2A, B, 3A, 4A-B). Barium study of the upper GI tract series can be used to detect volvulus and distal passage of ingested oral contrast material into the duodenum (Figure 4B). Multidetector CT (Figures 2C-F, 3B-D, 4D-F) is performed in the setting of emergency and can help to confirm the rotation of the herniated stomach and locate the transition point. If the twist of the bowel is > 180 degrees, there is obstruction of the distal end of the stomach, leading to dilatation of the proximal stomach. CT can also detect the status of the mesenteric vessels and the viability of the wall of the stomach. Hypoenhancement or non-enhancement of the bowel wall signifies ischemia and warrants the radiologist to alert the surgeon. Delay in diagnosis can lead to various complications like bowel wall ischemia, perforation, mediastinitis due to contamination mediastinum with content of perforated bowel, and peritonitis due to intrabdominal perforation.^1^ Thus, both types of volvulus need to be assessed promptly and should be considered as surgical emergencies when they present with acute pain and treated accordingly. The management of asymptomatic patients is usually dictated by the severity of symptoms.

## Small Bowel Volvulus

 Midgut volvulus is the most common type of volvulus in children but can be seen in adults, too. When present in children, patients usually present in 1^st^ month of life with bilious vomiting.^3^ The major predisposing factor for midgut volvulus is malrotation. Due to abnormal fixation of the small bowel mesentery, it results in an abnormally short mesenteric root, which causes the small bowel to twist around its mesentery and causes obstruction and possibly ischemia of the bowel. In adults, it manifests mostly as chronic intermittent abdominal pain that resolves when the volvulus spontaneously reduces.^6,8^

**Figure 5:**
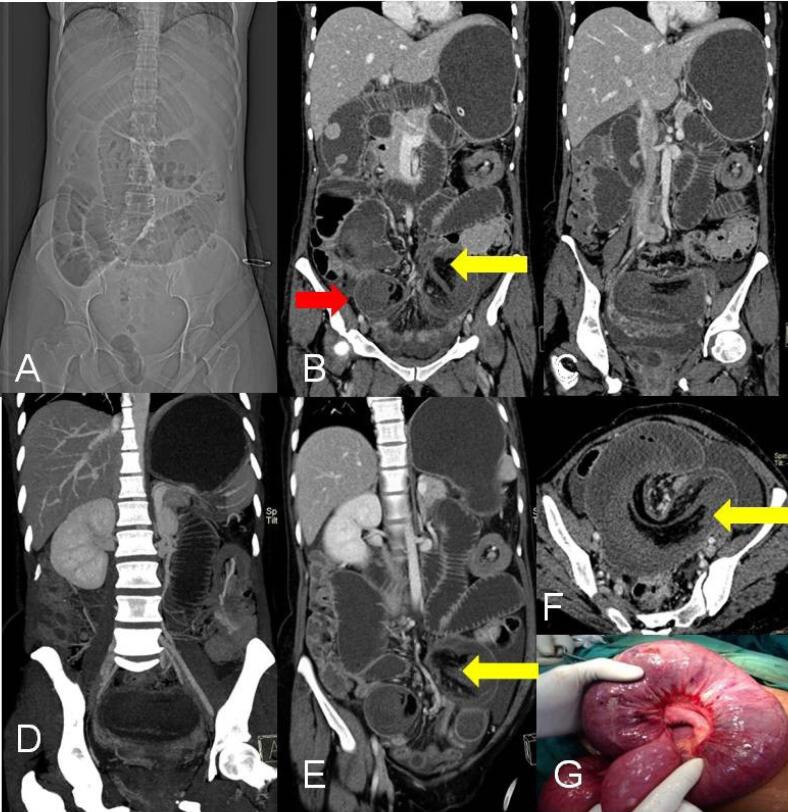
Small bowel volvulus: A young female presented with acute pain in the abdomen. The topogram shows multiple dilated small bowel loops. Coronal contrast-enhanced Multiplanar reformation and maximum intensity projection images show twisted ileal loops in the central lower abdomen (red arrow in image B) with proximal intussusception causing obstruction. The mesentery is also seen within the bowel lumen (yellow arrow in images B, E, F). Multiple homogenously enhancing polypoid lesions were seen in the bowel. The final diagnosis was that of small bowel obstruction secondary to volvulus and intussusception with polyposis syndrome. The intraoperative image (image G) shows constrictions of two adjacent bowel segments and the intervening mesentery forming a narrow pedicle leading to torsion of the loops

**Figure 6:**
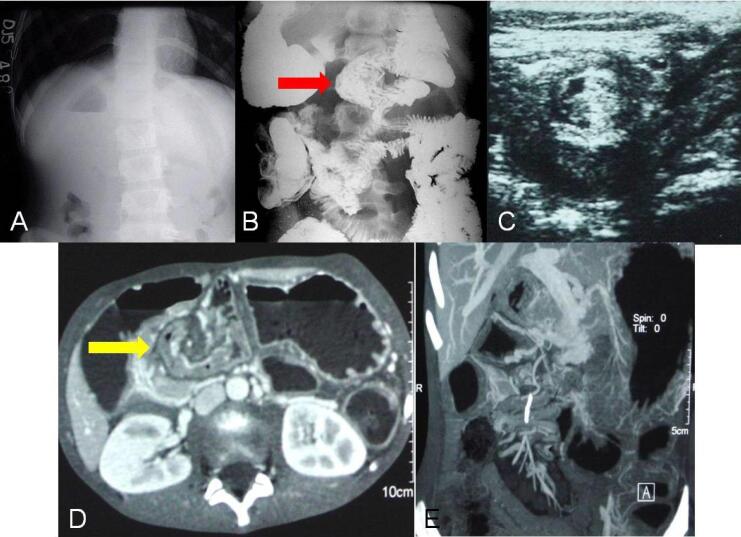
Midgut volvulus: A 13-year-old male patient presented with acute pain in the abdomen. A plain radiograph revealed the presence of the gastric fundus on the right side (the apex of the heart is towards left). Spot image of barium meal follow-through examination confirms the right-sided stomach and left-sided C loop of duodenum. The third part of the duodenum does not cross the midline (red arrow in image B). The jejunum, however, lies on the left side of the abdomen and the ileum on the right side. Ultrasoundfrom a linear probe shows swirling of bowel loops. The final diagnosis was situs inversus with malrotation and midgut volvulus. Axial contrast-enhanced tomogram in another patient, a 12-year-old girl with bilious vomiting, shows twisting of mesentery and reversal of superior mesenteric vessels (yellow arrow in image D) with proximal dilated bowel loops. Coronal maximum intensity projection images show the vessels in the twisted mesentery

**Figure 7:**
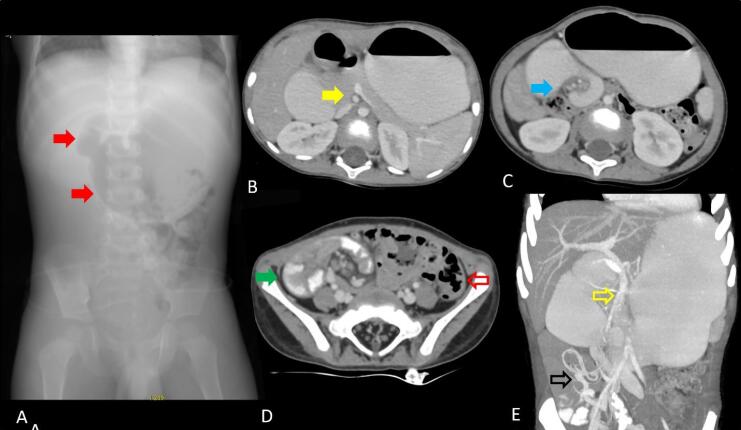
Midgut volvulus: A 3-year-old boy presented with acute pain in the abdomen. A plain radiograph revealed the presence of “double bubble sign” with a distended stomach bubble and duodenal bulb (red arrows in A). Axial contrast-enhanced tomogram confirms the twisting of mesentery (yellow arrow B) and reversal of superior mesenteric vessels (blue arrow in image C) with the small bowel located in the right hemiabdomen (green arrow in D) and large bowel located in left hemiabdomen (red hollow arrow in D). Coronal maximum intensity projection images show the vessels in the twisted mesentery, and the superior mesenteric artery is seen as a yellow hollow arrow. Multiple collaterals are seen in the mesentery (black hollow arrow) as superior mesenteric vein was compressed within the volvulus. The final diagnosis was malrotation with midgut volvulus

 Plain radiographs are either normal early on or have an appearance of bowel obstruction (Figure 5A). In children, special attention should be given to the location of abdominal organs, heart in chest on abdominal radiographs to look for signs of malrotation. An abnormal situs is indicated by the opposite location of the stomach bubble in relation to the apex of the heart with the contralateral location of the liver (Figure 6A). In neonates, a “Double bubble sign” can be seen due to a distended stomach and duodenum if the obstruction is complete (Figure 7A). On the fluoroscopic upper GI and small-bowel studies, there are characteristic signs due to malrotation i.e. abnormal position of most of the small bowel in the right abdomen and the third part of the duodenum is not seen to cross the midline, usually below and to the right of the left L1 pedicle, due to abnormal location of ligament of trietz. If midgut volvulus is present, the twisted proximal small bowel gives a characteristic corkscrew-like appearance in a fluoroscopic study (Figure 6B). Ultrasonography is helpful in determining the abnormal positional relationship between the superior mesenteric vein and artery (vein located to the left of the artery, opposite of usual orientation) (Figure 6C), but its drawback is that it does not provide a detailed evaluation of the bowel. At contrast enhanced computed tomography (CECT)swirling of vessels (Figure 5B-F, 6D-E, 7B) in the mesenteric root is seen at the site of the volvulus. CT also allows detailed evaluation of the bowel, especially to look for signs of malrotation like change in superior mesenteric vessel relationship to anteroposterior or complete reversal (Figure 7C) with abnormal location of -duodenojejunal flexure at the midline to the opposite side of the gastric fundus, along with abnormal placement of small and large bowel loops (Figure 7D) and ischemia like abnormal enhancement or non-visualization of the bowel wall with or without omental infarcts. Maximum intensity projection images help to assess the status of superior mesenteric vessels within the volvulus (Figure 5D, 7E).^1,8^

## Cecal Volvulus

 Cecal volvulus accounts for ~10% of all intestinal volvulus.^1^ They occur in comparatively younger patients ~30-60 years old, as compared with sigmoid volvulus. Congenital failure of bowel fixation to the retroperitoneum is a common predisposing factor as it allows the proximal colon to be free and mobile and thus prone to twisting. Acquired causes include those which cause restriction of the bowel at a fixed point, like an adhesion, abdominal mass or scarring from calcified lymph nodes, around which the bowel rotates.^1,2,9^ There are two types of cecal volvulus: (1) Cecum twists in the axial plane, there is rotation in a clockwise or counter-clockwise direction around the long axis, which relocates cecum in the right lower quadrant, and (2) Loop type of cecal volvulus, there is both twist and inversion such that the cecum occupies the left upper quadrant of the abdomen. The terminal ileum is also twisted along with the cecum. A gas-filled appendix can be seen. This variant is more prone to causing small bowel obstruction as the terminal ileum is also involved.^1,10^ There is another variant called “cecal bascule”. It occurs when the cecum folds anteriorly without any torsion due to its loose attachment to its mesentery. Some have also argued that it is a form of adynamic ileus.^1^

**Figure 8:**
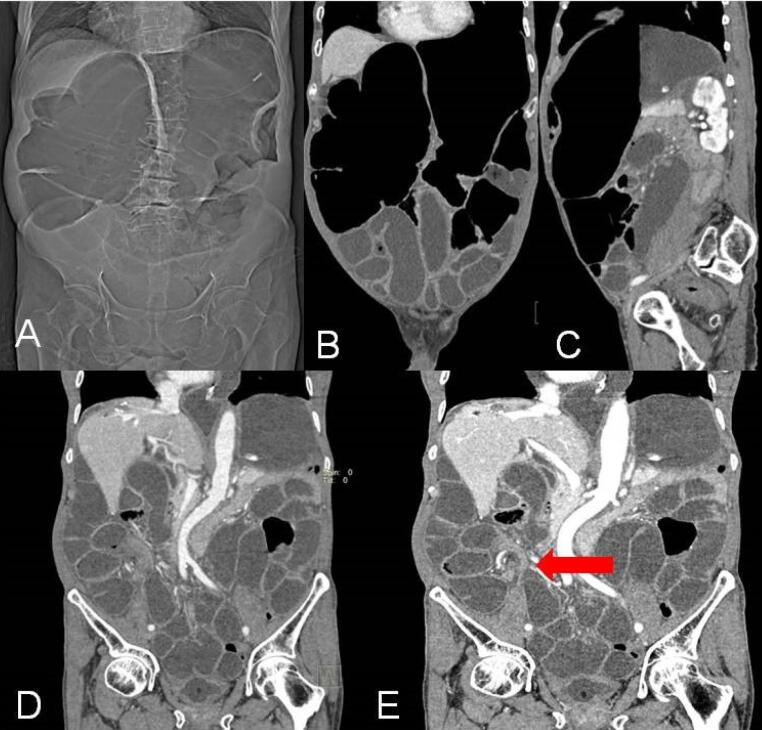
Cecal volvulus: An elderly male patient presented with a history of acute pain in the abdomen and distension. The topogram shows a large distended bowel loop with haustral folds in the epigastric region. The rest of the large bowel shows no air. Multiple coronal and sagittal contrast-enhanced CT sections show dilated bowel loops in the epigastrium extending from the region of the right lower quadrant. There is a twisting of mesentery in the right lower abdomen (red arrow in panel E)

**Figure 9:**
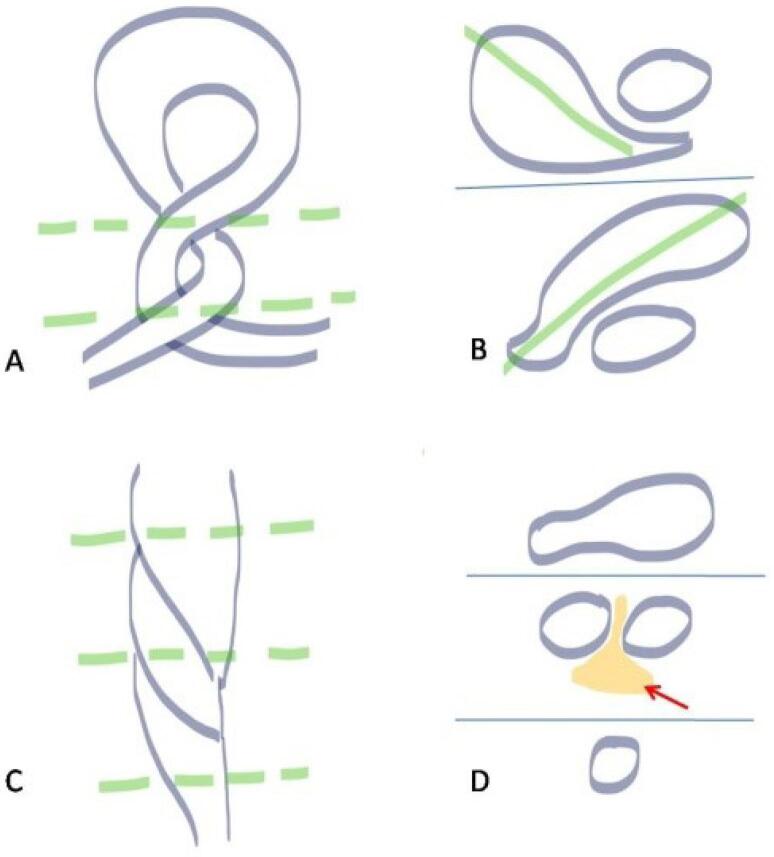
Illustration showing the X mark spot sign (images A, B) of complete cecal volvulus and split wall sign (images C, D) in incomplete cecal volvulus. Green dotted lines in images A and C mark the planes at which sections are shown in images B and D. The Red arrow in image D shows the mesenteric fat insinuating between the bowel walls

 The plain radiograph imaging of colonic volvulus is characteristic and often sufficient for diagnosis. There is marked distension of the large bowel loop. The long axis of the distended loop extends from the right lower quadrant to the epigastrium or the left upper quadrant with the caliber of the cecum (Figure 8A), often exceeding 9 cm. The obstruction is usually complete; hence, the distal colon is empty and the proximal small bowel is distended. During the contrast enema, the distal colon collapses, and there is beak-like tapering at the level of the volvulus. At CT, the cecum appears dilated and abnormally positioned in the upper mid and left abdomen, with the long axis of the dilated segment tracking back to the level of the volvulus where the classical whirl sign(Figure 8E) is seen. Few of the recently described imaging signs in cases of cecal volvulus include the X-marks-the-spot sign (Figure 9A, B), which is seen in cases of complete volvulus and refers to the crossing loops of bowel at the site of the transition. In cases of incomplete or recently resolved complete volvulus, a split wall sign (Figure 9C, D)is seen, which refers to mesenteric fat seen indenting or invaginating the wall of the bowel.^11^

## Transverse Colon Volvulus

 It is the rarest site of colonic volvulus seen in < 5% of cases ^1^ but is associated with the highest mortality. Abnormal fixation of the long mesentery of the transverse colon is the most common predisposing factor. Conventional radiography is not helpful. Contrast enema study shows the characteristic beakliketapering at the level of twist. The diagnosis is usually made on CT, which shows proximal bowel obstruction and the classic mesenteric swirl sign.

## Sigmoid Volvulus

**Figure 10:**
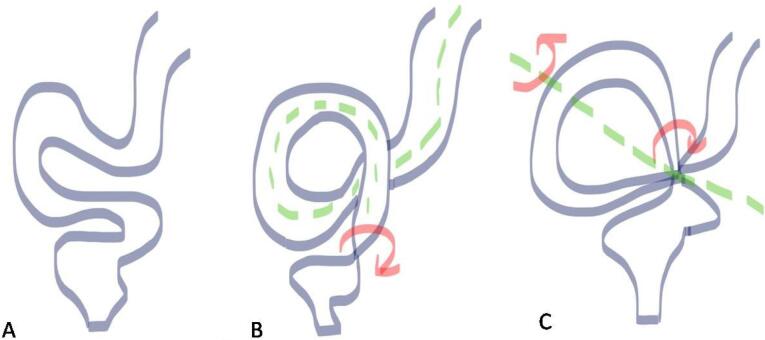
Diagrammatic representation showing normal sigmoid colon (A), organoaxial type (B) of sigmoid volvulus and mesenteroaxial type (C) of sigmoid volvulus

**Figure 11:**
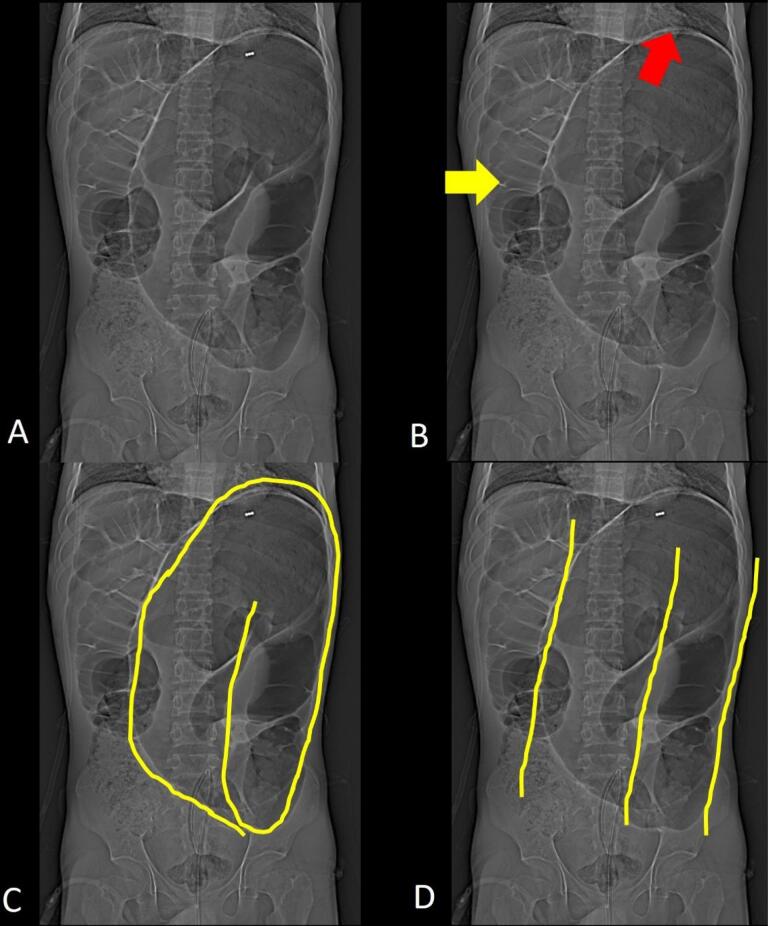
Sigmoid volvulus: An elderly male patient presented to the emergency room with a history of acute pain in the abdomen and constipation for a long time. The topogram shows a large dilated bowel loop extended cranially from the region of the left lower quadrant. It shows the classical northern exposure sign (red arrow in B), coffee bean sign (yellow lines in C), and three-line sign (yellow lines in D)

**Figure 12:**
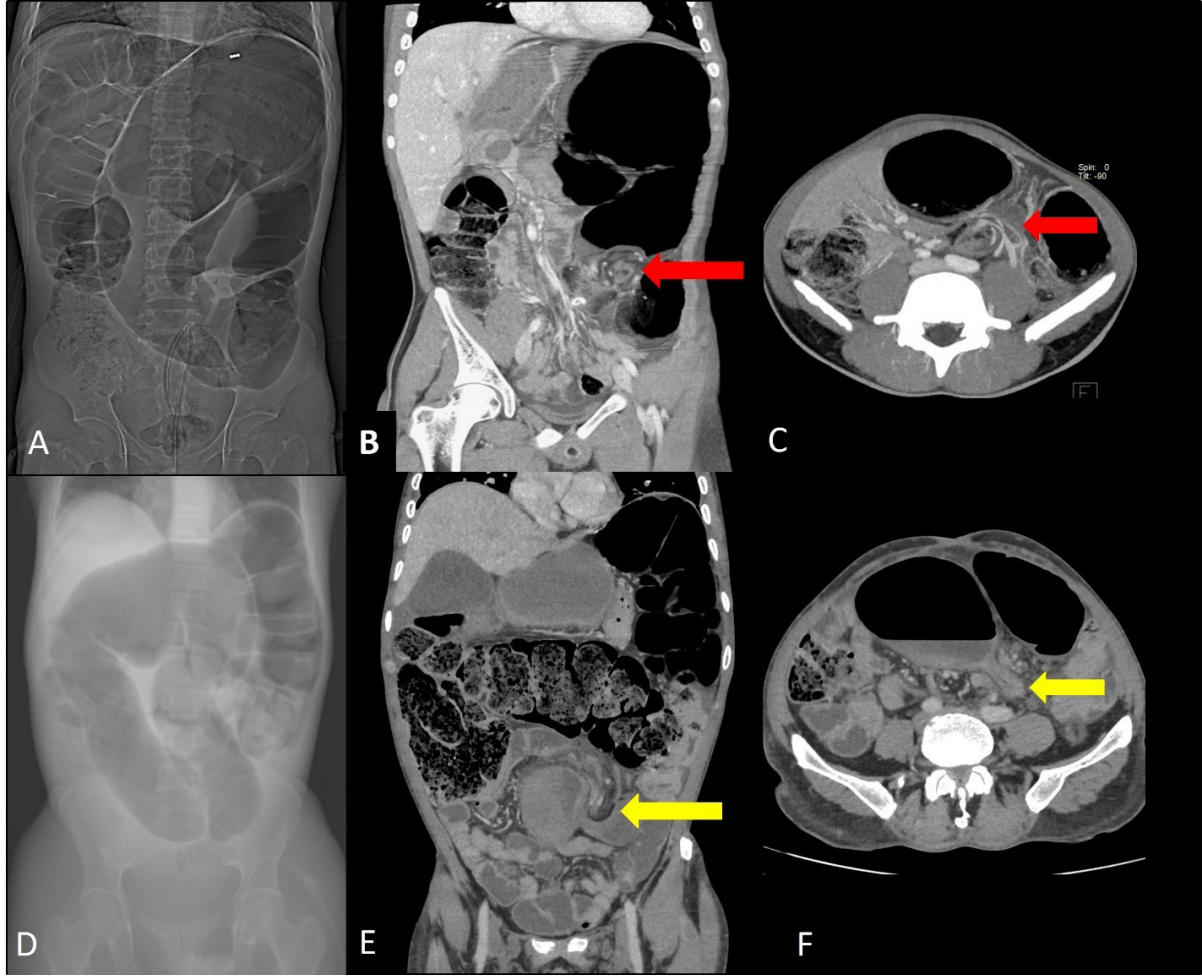
Sigmoid volvulus (mesenteroaxial type): Topogram of the previous patient (A) along with MPR coronal and MIP axial contrast-enhanced sections of CT of the same patient shows the classical whirl sign (red arrow in images B and C). Intraoperatively long mesentery of the sigmoid colon was found to be the cause of volvulus, and hence it was fixated after de-rotation. For another 55-year-old male patient with acute onset pain in the abdomen, an abdominal radiograph showed a grossly dilated bowel pointing to the right hemidiaphragm and with a base in the left iliac fossa. Coronal and axial sections of contrast-enhanced CT show a whirl sign (yellow arrows in E and F) located in the left iliac fossa, confirming sigmoid volvulus. Intraoperatively, adhesions from the presacral region to the sigmoid colon were found to be the cause of volvulus.

 The sigmoid is the most common site of colonic volvulus and accounts for 60-75% of all cases of colonic volvulus.^1^ It is generally considered to be an acquired condition which occurs in old age because its prevalence increases among those with chronic constipation and sigmoid colonic redundancy secondary to high fiber diet, pregnancy, hospitalization or Chagas disease. It is of two types: organoaxial volvulus and mesenteroaxial volvulus (Figure 10B, C).^2^ Plain radiographic findings diagnostic of sigmoid volvulus include a large air-filled bowel loop arising from the pelvis and extending cranially beyond the level of the transverse colon called the northern exposure sign (Figure 11B).^12^ Other features include the coffee bean sign i.e. coffee bean-like shape that the dilated sigmoid colon (Figure 11C).^13^ The closed-loop sign, which describes the U-shaped closed-loop appearance of the colon dilated between the two points of obstruction at the site of the volvulus; white-stripe sign, the obliquely oriented vertical white lines that represent the opposed walls of the dilated bowel loop (the center line) and the outer walls of the bowel loop on either side i.e. three-line sign (Figure 11D).^1,14^ In an enema study, the beak sign is seen as similar to the cecal volvulus. It may also help to achieve a reduction of the volvulus. At CT, the signs are similar to cecal volvulus (Figure 12 B-C). The points of differentiation between the sigmoid and the cecal volvulus are highlighted in Table 3.

**Table 3:** Differences between imaging of cecum and sigmoid volvulus

	**Cecal volvulus**	**Sigmoid volvulus**
Long axis of dilated bowel segment	Points from the right lower quadrant to the epigastrium or left upper quadrant	Points from the left lower quadrant to the epigastrium or right upper quadrant.
Colonic haustra	Maintained	Effaced
Air fluid levels	Single	Multiple

## Treatment

 Treatment of any volvulus usually requires emergent surgery with de-rotation of the twisted bowel and removal of the ischemic bowel within two days. If there is no ischemia, surgical fixation of the involved bowel is the treatment of choice, which might result in recurrence in future. In the case of sigmoid volvulus, conservative treatment like sigmoidoscopy or a barium enema can be undertaken initially.^1,2^

## Conclusion

 Volvulus can affect all segments of the bowel and is considered a surgical emergency; hence, it requires early diagnosis. It is difficult to diagnose them solely based on clinical features, as they are often non-specific and may include acute onset abdominal pain associated with vomiting. The radiologist is usually the first to diagnose a volvulus based on characteristic imaging findings. Thus, knowing classical imaging findings of these conditions helps us to make prompt diagnoses and avoid gruesome complications like bowel ischemia, infarction and perforation.
